# Geographic Mosaics of Fly Pollinators With Divergent Color Preferences Drive Landscape-Scale Structuring of Flower Color in Daisy Communities

**DOI:** 10.3389/fpls.2021.617761

**Published:** 2021-02-01

**Authors:** Allan G. Ellis, Bruce Anderson, Jurene E. Kemp

**Affiliations:** Department of Botany and Zoology, Stellenbosch University, Matieland, South Africa

**Keywords:** Asteraceae (compositae), Diptera, flower color, geographic mosaics of pollinators, greater cape floristic region, pollinator driven divergence, sensory drive, South Africa

## Abstract

The striking variation in flower color across and within Angiosperm species is often attributed to divergent selection resulting from geographic mosaics of pollinators with different color preferences. Despite the importance of pollinator mosaics in driving floral divergence, the distributions of pollinators and their color preferences are seldom quantified. The extensive mass-flowering displays of annual daisy species in Namaqualand, South Africa, are characterized by striking color convergence within communities, but also color turnover within species and genera across large geographic scales. We aimed to determine whether shifts between orange and white-flowered daisy communities are driven by the innate color preferences of different pollinators or by soil color, which can potentially affect the detectability of different colored flowers. Different bee-fly pollinators dominated in both community types so that largely non-overlapping pollinator distributions were strongly associated with different flower colors. Visual modeling demonstrated that orange and white-flowered species are distinguishable in fly vision, and choice experiments demonstrated strongly divergent color preferences. We found that the dominant pollinator in orange communities has a strong spontaneous preference for orange flowers, which was not altered by conditioning. Similarly, the dominant pollinator in white communities exhibited an innate preference for white flowers. Although detectability of white flowers varied across soil types, background contrast did not alter color preferences. These findings demonstrate that landscape-level flower color turnover across Namaqua daisy communities is likely shaped by a strong qualitative geographic mosaic of bee-fly pollinators with divergent color preferences. This is an unexpected result given the classically generalist pollination phenotype of daisies. However, because of the dominance of single fly pollinator species within communities, and the virtual absence of bees as pollinators, we suggest that Namaqua daisies function as pollination specialists despite their generalist phenotypes, thus facilitating differentiation of flower color by pollinator shifts across the fly pollinator mosaic.

## Introduction

Flower color diversity is a striking feature of the angiosperms, with closely-related plant species, and populations within species, frequently varying geographically in flower color or patterning (e.g., Cooley et al., 2008; Ellis and Johnson, 2009; Newman et al., 2012; Muchhala et al., 2014; Wang et al., 2016). Spatial divergence in flower color can arise through neutral processes (Rausher, 2008; Wang et al., 2016; Koski and Galloway, 2020), or as a response to selection imposed by abiotic conditions (Koski and Ashman, 2015; Dalrymple et al., 2020) or non-pollinating flower visitors (Strauss and Whittall, 2006; de Jager and Ellis, 2014b; Kemp and Ellis, 2019). Alternately, under the pollinator-shift or Grant-Stebbins model of divergence, geographic mosaics of pollinators with different morphology or sensory systems can drive the divergence of floral traits, which may ultimately facilitate speciation (Johnson, 2006).

The broad associations between flower color and different pollinator groups offer indirect evidence that pollinators may have played a crucial role in floral color evolution (Fægri and van der Pijl, 1966; Fenster et al., 2004), while selection studies demonstrate more directly that pollinators can select on flower color (Harder and Johnson, 2009; Sletvold et al., 2016). Furthermore, geographically structured color forms of the same species (floral ecotypes), or closely related species, are frequently visited by different pollinators, providing additional evidence that pollinators are important drivers of flower color divergence (e.g., van der Niet et al., 2014; Newman et al., 2015; Kemp et al., 2019). However, one weakness of many associative studies between color and pollinator shifts is that the pollinator gradients which power floral divergence are rarely quantified independently of the focal plant taxa. Pollinator abundances are usually quantified through observations of visits to the focal flowers, with only a few studies employing independent datasets, such as pollinator distribution records (e.g., Waterman et al., 2011; van der Niet et al., 2014) or trapping surveys (e.g., Phillips et al., 2015), to quantify pollinator gradients. Consequently, it is seldom clear whether geographic associations between floral phenotype and pollinator assemblages are the evolved plant responses to underlying spatial mosaics of pollinator availability, or the result of spatially variable outcomes of competitive interactions of co-occurring flowers for pollination services (Muchhala et al., 2014).

Spatial pollinator mosaics can be qualitative, where different pollinators are either present or absent in different parts of the landscape, as envisaged by the classic Grant-Stebbins model (e.g., van der Niet et al., 2014). Pollinator-shift mediated floral divergence across qualitative gradients is most likely when plants have somewhat specialized pollination systems (Johnson, 2010). In contrast, spatial mosaics underlying divergence in generalist plants tend to be quantitative, involving subtle variation in the relative availability (density) of the same assemblage of pollinators across the landscape (Dilley et al., 2000; Gómez et al., 2009, 2014). Whether qualitative or quantitative, flower color divergence is expected across pollinator gradients when the dominant pollinators exhibit contrasting flower color preference resulting from innate differences in visual systems and sensory biases. This may frequently be the case, given the diversity of color vision systems across pollinating animals (Briscoe and Chittka, 2001; van der Kooi et al., 2021). However, associative learning, which is widespread in pollinators (Chittka et al., 1999; Dukas, 2008), can overwrite innate color preferences or interact with them in complex ways (Giurfa et al., 1995; Giurfa, 2004; Dyer et al., 2019). The resulting flexibility in flower color preferences and floral constancy could potentially reduce the strength of divergent selection on flower color across pollinator gradients (de Jager et al., 2011), or in some cases even generate divergent selection on flower color (Newman et al., 2012) if learned preferences are stable enough in time.

As detectability of flowers is dependent on contrast with background colors (e.g., leaves or soils; Bukovac et al., 2017; Koski, 2020), flower color divergence might also occur across spatial gradients in background coloration, such as geological boundaries. Thus, pollinators might favor flower colors that are more detectable in a particular environment, rather than choosing a particular color *per se*. This may be particularly important for flowers that are presented against soil backgrounds, such as in deserts (Menzel et al., 1997), where the background color is determined by spatially varying geologies. While the influence of background contrast on flower color evolution is a clear expectation of the sensory drive theory of signal evolution (Endler, 1992), it remains largely untested (Bukovac et al., 2017; Koski, 2020). Under this model, divergence in flower color should be underlain by spatial turnover in background color, but not necessarily turnover in available pollinators. The same pollinator, or pollinators with similar visual systems, could select for the most detectable flower colors across spatial gradients in background coloration, resulting in flower color divergence.

**Figure 1:**
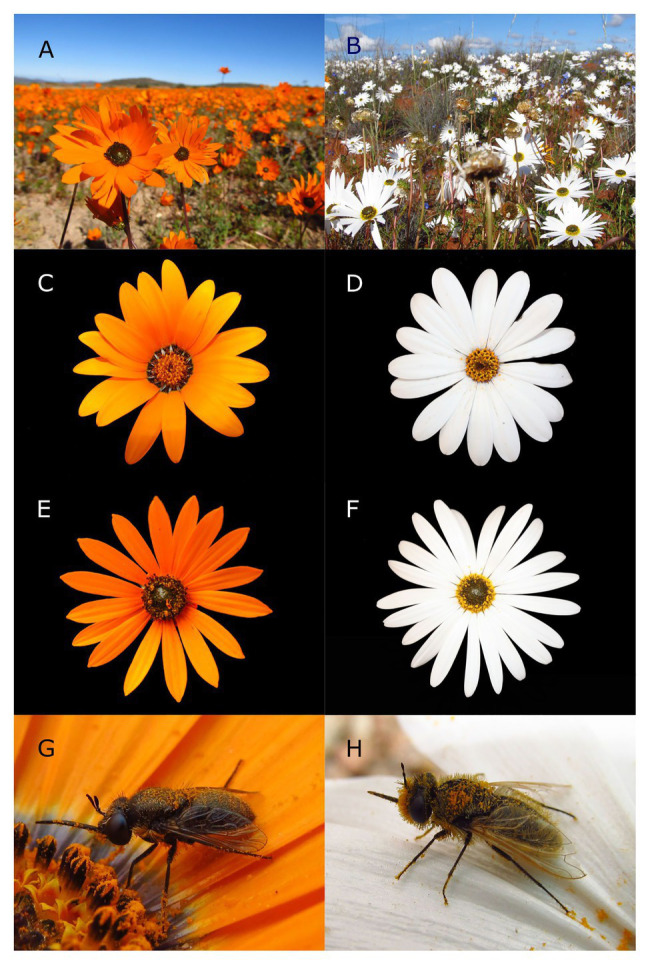
The annual daisy species and their pollinators that characterize orange **(A)** and white **(B)** dominated communities in Namaqualand. *Dimorphotheca sinuata* **(C)** and *Dimorphotheca pluvialis* **(D)** are widespread and dominant in the Kamiesberg and Sandveld bioregions, respectively. *Ursinia cakilefolia* **(E)** and *Ursinia speciosa* **(F)** are more localized but dominate communities they occur in. The bombyliid flies, *Megapalpus capensis* **(G)** and *Corsomyza nigripes* **(H)** are dominant pollinators in the orange and white communities, respectively. The inflorescence diameter of the two *Dimorphotheca* species is on average 35mm, and the diameter of the two *Ursinia* species is on average 31mm. Both fly species show variation in size but are always smaller than 10mm. Photos: JEK.

Here we focus on the striking landscape-level structuring of dominant flower colors in the spectacular spring mass-flowering displays of annual daisies in the arid Namaqualand region of the greater cape floristic region (GCFR) in South Africa (Figure 1). Kemp et al. (2019) recently showed convergence of dominant species within flowering daisy communities on shared flower color patterns. In addition, dominant flower color patterns shift across broad spatial scales, often across geological boundaries, and shifts usually involve multiple floral ecotypes or closely related species. Analysis of daisy (the dominant flowering family in these communities) visitation networks revealed that shared flower color patterns within and across communities are strongly associated with visitation by different bombyliid and tabanid flies (Kemp et al., 2019). This suggests that the spatial color structuring of mass flowering displays across the landscape might arise through spatially variable selection on flower color, or ecological filtering on the basis of flower color, across spatial gradients in the availability of the dominant fly pollinator species. However, little is known about distributions of these pollinators, and thus the structure of the geographic pollinator mosaic that underlies the landscape structuring of flower color.

The visual systems and color preferences of Namaqualand fly pollinators are also unknown, although one of the important bombyliid pollinators, *Megapalpus capensis* (Wiedemann), does exhibit strong behavioral responses to floral visual signals (Johnson and Midgley, 1997; de Jager and Ellis, 2012, 2014b). This knowledge gap is not surprising, given the limited understanding of color vision and preferences of flower-visiting flies generally (Lunau, 2014; Schnaitmann et al., 2020; van der Kooi et al., 2021). However, extensive variation in flower color associations (e.g., Goldblatt and Manning, 2000; Shrestha et al., 2016), photoreceptor sensitivities (van der Kooi et al., 2021), and color preferences in contexts other than flower visitation (Lunau, 2014), suggests that flower-visiting flies likely exhibit a diversity of color vision systems and innate color preferences (Lunau, 2014; van der Kooi et al., 2021), and that these may differ from those of bees (Shrestha et al., 2019).

We studied the dominant fly pollinators of focal species pairs in the daisy genera, *Dimorphotheca* and *Ursinia*, which are abundant in Namaqualand annual daisy communities dominated by white or orange flowers. We use community-level surveys to quantify the density of these pollinators across the landscape and test whether the flower color of the focal daisy species is predicted by the underlying fly density gradients. We then use visual modeling to test distinguishability and detectability of flower colors in fly vision, and cage choice experiments to test for divergent color preferences and for the influence of soil coloration on the detectability of orange and white flowers.

We investigated two potential explanations for the landscape level structuring of daisy flower color. First, if the geographic structure of flower color results from pollinator shifts across a geographic pollinator mosaic, we expect: (1) that dominant pollinator species should exhibit geographic mosaics of availability (density), (2) that pollinator species distributions should be associated with flower color distributions, and (3) that pollinators should exhibit divergent color preferences that are not flexible. Second, if the geographic structure of flower color arises through pollinator-mediated selection for detectability of flower colors across a mosaic of chromatically different soil backgrounds, we expect: (1) that flowers should be most detectable to pollinators on their local soil background and (2) that pollinator flower color preferences might be altered by the background color.

## Materials And Methods

### Study System

Our study was conducted in the Namaqualand region of the Succulent Karoo, the arid component of the hyper-diverse GCFR (Born et al., 2007). We focused on two bioregions as defined by Desmet (2007): the Kamiesberg that consists of granite-gneiss dome-shaped hills, with variably grained pale soils, and the Sandveld that comprises marine-derived sands that are often red in color. In the Kamiesberg annual spring-flowering daisy displays are orange (Figure 1A), while the Sandveld flowering displays of annual daisies are dominated by white-flowered species (Figure 1B; data from Kemp et al., 2019). We focus on white-orange species pairs of *Dimorphotheca* and *Ursinia*, two distantly related Asteraceae genera (from the Calenduleae and Anthemideae tribes, respectively). *Dimorphotheca* species dominate the Namaqualand annual displays and are widespread, while *Ursinia* species are less widespread but dominate displays at some sites. Each pair comprised one species with large white ray florets [i.e., *Dimorphotheca pluvialis* (L.) Moench and *Ursinia speciosa* DC., Figure 1] and one with orange ray florets (i.e., *Dimorphotheca sinuata* DC. and *Ursinia cakilefolia* DC., Figure 1). For convenience, we refer to these as white and orange “flowered” species, respectively. While phylogenetic relationships in *Ursinia* are not resolved, *U. speciosa* and *U. cakilefolia* are members of the same clade in subgenus *Ursinia* (Magee et al., 2014). *Dimorphotheca pluvialis* and *D. sinuata* are very closely related, producing hybrids in cultivation, and the only consistent phenotypic difference between these two species is their flower color (Norlindh, 1943). By using phylogenetically closely related species pairs, we minimize potential chemical or morphological factors that might differ between species.

Few ecological studies have been conducted on the pollinators of annual Namaqualand daisies, and work has mostly focused on the bee fly *M. capensis* that is the dominant pollinator of the hypervariable, sexually deceptive daisy, *Gorteria diffusa* (Ellis and Johnson, 2009, 2010, 2012; de Jager and Ellis, 2012, 2013, 2014a). This fly responds strongly to floral visual signals (de Jager and Ellis, 2012, 2014b) and is the dominant visitor to several species of the orange-flowered daisies which can dominate mass-flowering displays (e.g., *Dimorphotheca pinnatum* – de Waal et al., 2015, *Ursinia calenduliflora* – de Jager and Ellis, 2014b). Analysis of floral visitation networks (Kemp et al., 2019) demonstrated that, while annual Namaqualand daisies are visited by a wide array of insects as expected from their generalist phenotype, visitation is dominated by small fly species (Bombyliidae, Tabanidae), each associated with different flower color patterns across communities.

### Are Flower Color Distributions in *Dimorphotheca* and *Ursinia* Overlapping?

The focal species of *Dimorphotheca* and *Ursinia* were encountered at 54 sampling sites across Namaqualand. At each site, the density of inflorescences of each species was quantified in at least twenty 1m^2^ plots along transects across a 100 × 100m sampling area. To assess the spatial distribution of flower color, communities containing the focal species were mapped as comprising orange only, white only, or both flower colors.

### Are Different Flower Colors Visited by Different Pollinator Species?

To assess whether orange and white flowers were visited by different pollinator species, we sampled 59 populations of the focal *Dimorphotheca* and *Ursinia* species in austral spring 2013–2015. We walked multiple transects through a 100 × 100m sampling area at each site and recorded the identity and abundances of all insect species present in inflorescences of the focal plant species (mean = 690 inflorescences sampled per population, range 111–2,074). We calculated the number of individuals per flowerhead for each insect species to identify the dominant pollinator species on white and orange-flowered species, and we will focus on these dominant pollinators for the rest of the paper. We identified the dominant pollinators using two criteria: (1) species that were consistent visitors in space (i.e., present as pollinators across the most sites) and (2) species that were most frequent visitors (i.e., species that contributed the highest proportion of visits at sites).

### To What Extent Do the Distribution Ranges of Dominant Pollinators Overlap?

The densities of these dominant fly pollinators were quantified using surveys at 103 sites across Namaqualand over 4years (2013–2016) during austral spring. First, at each site (ca. 100 × 100m) we walked multiple transects and surveyed flowers/flowerheads of all plant species on which individuals of the target pollinator species were observed, and from this, we calculated the number of insect individuals per flower/flowerhead for each plant species. This included the four focal daisy species when present, but also numerous other species of daisy, and members of other families, such as the Aizoaceae. We then estimated flower/flowerhead densities of each plant species in at least twenty 1m^2^ plots sampled on transects across the sites, and from this, we could estimate the abundances per square meter of both fly species at each site. This approach provided an estimate of availability (i.e., density) of each pollinator species independent of the phenotype of the focal plant species. To assess the spatial distribution of the identified dominant pollinators, communities were mapped as comprising *M. capensis* only, *Corsomyza nigripes* Wiedemann only, or both pollinator species.

### Are Flower Color Distributions Associated With Pollinator Availability?

If pollinator species occurrence predicts flower color distributions, we expect that the dominant flower color in a community should be predicted by the availability (density m^−2^) of pollinator species. To test this, we used the fly density and flower color data from the 54 sites where our focal plant species occurred. We coded site-level flower color, the response variable, as a binary variable (i.e., assigning orange as “1” and white as “0”) because orange and white flowers did not co-occur at any sites. We ran two separate logistic regression models testing whether (1) the presence of orange flowers at a site was predicted by *M. capensis* density and (2) whether the presence of white flowers was predicted by *C. nigripes* density. As the model using *C. nigripes* densities did not converge because of complete separation (i.e., orange flowers were only present when *C. nigripes* was absent), we used Firth’s bias-reduced logistic regression as implemented in the R-package logistf (Heinze and Ploner, 2018).

### Are Flower Colors Detectable and Distinguishable in Fly Vision?

The reflectance spectra of the ray florets of the study species were recorded using an Ocean Optics USB4000 spectrometer, following the protocol outlined in Kemp et al. (2019). Spectra were averaged across three to five separate capitula of each species. Soil color varies strikingly across the range of our focal plant taxa, with white flowers usually presented against red marine-derived sand and orange flowers against pale yellow granite gneiss derived soil. Because background contrast can influence the detection abilities of insects (Bukovac et al., 2017), we modeled flower color in fly vision against both these background types, using a multispectral imaging approach. This was necessary because the fine granular structure of sand prevented us from reliably quantifying spectra of the sand/soil substrates using spectrometry. Four to five inflorescences of each species were imaged on each of the soil backgrounds (i.e., pale granite and red sand) using a converted and calibrated Nikon D7100 camera and an AF-S Micro Nikkor 105mm f/2.8 lens that transmits UV wavelengths (Troscianko and Stevens, 2015). Photographs were taken in diffused sunlit conditions using a Baader UV/IR blocking filter, that transmits from 400 to 700 nm, and a Baader U-filter optimized for transmission in the 325–369nm range. Images were processed using the MICA toolbox (v1.22) for imageJ (v.1.4.9; Troscianko and Stevens, 2015). First images were calibrated against Spectralon 20 and 80% reflectance standards to control for lighting conditions, and then the visible and UV images were combined into aligned, normalized multispectral stacks.

Photoreceptor quantum catches were calculated for the visual system of the flower-visiting syrphid fly, *Eristalis tenax* (An et al., 2018), using the cone mapping function in the MICA toolbox. Photoreceptor sensitivities are not known for any bombyliid flies (van der Kooi et al., 2021). Quantum catches were calculated for five regions on the ray florets and soil backgrounds in each image, which were then averaged for each image. Photoreceptor excitation values for ray florets in each image were then modeled, using the color-opponent coding fly vision model of Troje (1993), against the background quantum catch for that image. Modeling followed Ohashi et al. (2015) under D65 standard daylight illumination.

The Troje (1993) model, based on data from *Lucilia* blowflies, posits that fly color vision is based on two opponency mechanisms involving two pairs of photoreceptors that allow flies to distinguish four color categories dependent on the relative excitation of these paired photoreceptors. Distinguishable color categories are represented by the four quadrants in categorical fly visual space, while colors within quadrants are not distinguishable under the Troje (1993) model. However, experiments with the flower-visiting syrphid fly, *E. tenax*, suggest that color processing is not categorical and that these flies can discriminate fine-scale differences in color within quadrants of fly color space (Hannah et al., 2019). Consequently, we used Euclidean distances from the origin of fly color space to assess the detectability of flowers on different background substrates in relation to the minimum color discrimination thresholds determined experimentally by Hannah et al. (2019). Welch’s *t*-tests were used to determine whether Euclidean distances of each species from the origin of fly color space (i.e., detectability) were greater on native soils. While Hannah et al. (2019) only considered minimum discrimination distances against a background color (i.e., Euclidean distance from the origin of fly color space) and not discrimination of colors, we cautiously use these thresholds as a guide to assess discriminability of different flower colors in the color space (quantified as Euclidean distances between species centroids).

### Do Pollinators Have Divergent Flower Color Preferences?

We conducted a series of choice experiments to determine flower color preferences of the dominant pollinator taxa (i.e., *M. capensis* in orange communities and *C. nigripes* in white communities) and the influence of soil background color on preferences. Wild-caught fly individuals were presented with choices between the orange and white *Dimorphotheca* and *Ursinia* species pairs separately.

To account for the potential influence of background contrast on color preference, we used the two different soil types (i.e., red sand and pale granite soil) as background for the flowers during the choice experiments. Experimental arenas contained both soil types with eight inflorescences (four orange and four white inflorescences from the same genus) placed 15cm apart in eppendorf tubes on each soil type (i.e., 16 inflorescences in total; see Supplementary Figure S1). Individual flies of each pollinator species were released separately into the caged arenas, and allowed to sequentially visit inflorescences. The flower color and soil background were recorded for each visit. Trials were terminated after 5min or when a fly had made 20 choices (median number of choices was five). Inflorescences were replaced every five trials to avoid nectar depletion. Individual flies were tested on both the *Dimorphotheca* and *Urisinia* species pairs in random order.

To test for significant differences in flower color preference between fly species, we ran a Generalized Estimating Equation (GEE) which allowed us to control for non-independence of individual fly choices. Flower color choice was set as the binomial response variable, and fly species, soil type, and their interaction were used as predictors. The model assumed an exchangeable correlation structure where the sequential choices of fly individuals are equally correlated. A binomial distribution with a logit link function was used to obtain the estimated marginal means and their 95% confidence intervals based on approximate jackknife variance estimates. Models were run separately for *Dimorphotheca* and *Ursinia* choices. Fly individuals that did not visit flowers on both soil types were excluded from the data set (*Corsomyza*: 3 of 45 trials were excluded; *Megapalpus*: 6 of 93 trials were excluded). All analyses were done in R (R Core Team, 2020) and GEEs were conducted using the geepack package (Halekoh et al., 2006).

A second set of binary choice experiments were conducted to directly assess the influence of soil background color on flower detectability. Individual insects were offered a choice between inflorescences of the same species presented on two different soil types (four inflorescences on pale soil and four on red sand). *Megapalpus capensis*, the dominant pollinator in orange communities, chose between orange *D. sinuata* flowers presented on different soils, while *C. nigripes*, the dominant pollinator in white communities, chose between white *D. pluvialis* flowers. Only the first choice of each fly was recorded, and inflorescences were replaced after every five trials. To determine whether background (i.e., soil type) influenced pollinator flower choices, Chi-square tests, expecting no differentiation in choice frequencies between soil types, were conducted separately for each fly species.

The flower color preferences of the wild-collected flies in the previous experiments could reflect either innate preferences or conditioning through experience with the particular flower color with which they associate in the field (and lack of experience with the alternative color). To distinguish between these possibilities, we conducted a series of absolute conditioning experiments (Lunau et al., 2018) to determine whether color preferences could be altered through conditioning. In the first experiment, flies were forced to feed exclusively on arrays comprising fresh inflorescences of both species of their non-preferred color (i.e., white for *M. capensis* and orange for *C. nigripes*). After an hour of conditioning, during which flies were observed to feed copiously on both nectar and pollen, their color preference was again determined using the color choice experiment described previously. In a second experiment, *M. capensis* individuals were conditioned on the non-preferred flower color (white) for a full day before re-testing. Both *Dimorphotheca* and *Ursinia* species pairs were used for the *C. nigripes* conditioning experiment, but only *Dimorphotheca* inflorescences were available for the *M. capensis* experiments. Separate GEEs, with a binomial distribution and logit link function, were used to determine whether the flower color choice of each fly species was altered by conditioning.

## Results

### Are Flower Color Distributions in *Dimorphotheca* and *Ursinia* Overlapping?

**Figure 2:**
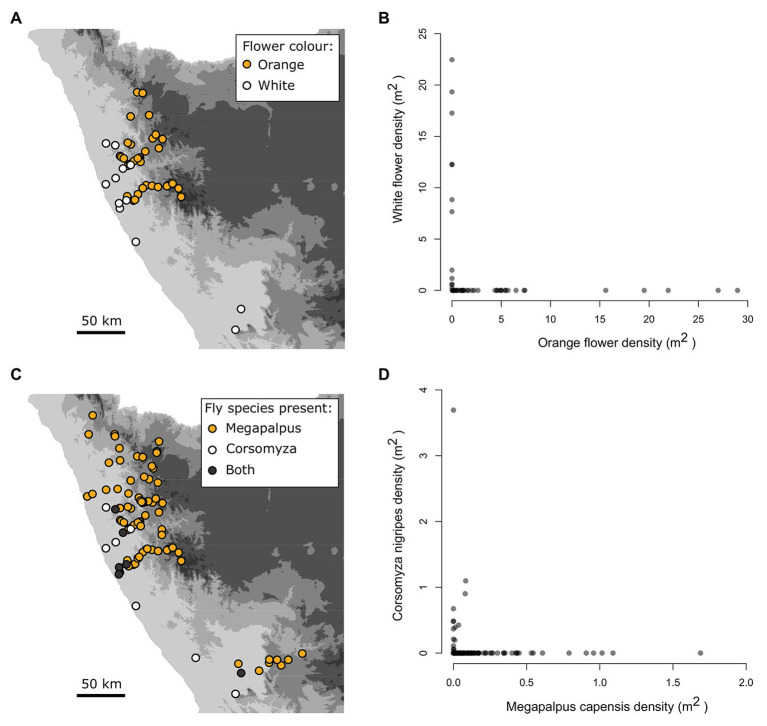
Strongly aligned spatial gradients of flower color and pollinators of annual daisy species across the Namaqualand landscape. The non-overlapping distribution **(A)** and density relationships **(B)** of the focal orange (*Dimorphotheca sinuata*, *Ursinia cakelifolia*) and white (*Dimorphotheca pluvialis*, *Ursinia speciosa*) flowered species at 54 sites. The geographic mosaic **(C)** and density relationships **(D)** of the dominant bee-fly pollinators, *Megapalpus capensis*, and *Corsomyza nigripes*, across 103 survey sites.

Flower color was entirely non-overlapping at the 54 sites where the focal plant taxa occurred (Figures 2A,B. The white species were only present in the Sandveld region, while the orange species were present in the Kamiesberg region (Figure 2A).

### Are Different Flower Colors Visited by Different Pollinator Species?

Two different fly pollinators, *M. capensis* and *C. nigripes* (Figure 1), both from the bombyliid subfamily Mariobezziinae, were identified as the dominant pollinators of orange and white daisies, respectively. *Megapalpus capensis* contributed 45% of all observed visits on orange flowers and 1.2% on white flowers, while *C. nigripes* contributed 87% of visits to white flowers and was not observed on orange flowers (Table 1). Other pollinator species contributed substantially fewer visits, with bees being virtually absent (Table 1).

**Table 1:** Proportion of total visits contributed to focal white and orange daisies by the main groups of insect pollinators.

	White flowers	Orange flowers
*Corsomyza nigripes*	87.3%	0%
*Megapalpus capensis*	1.2%	45.0%
Other flies	1.4% (4)	19.2% (8)
Hopliini beetles	3.4% (7)	25.1% (21)
Other beetles (mainly Meloidae)	6.6% (3)	10.3% (10)
Bees	0.1% (1)	0.3% (2)

Dominant fly pollinators are *Megapalpus capensis* for orange daisies and *Corsomyza nigripes* for white flowers. Data are the percentage of all insects observed on 9,334 white and 31,402 orange inflorescences during walked surveys. Numbers in brackets are the number of pollinator species/morphospecies in each group.

*Megapalpus capensis* was both the most consistent pollinator on orange flowers (present at 92% of *D. sinuata* populations and 100% of *U. cakelifolia* populations, Table 2), and the most frequent visitor (contributing 50% of visits on *D. sinuata* and 37% of visits on *U. cakelifolia*). Similarly, *C. nigripes* was the most consistent pollinator on white flowers (present in 100% of *D. pluvialis* and *U. speciosa* populations) and most frequent on *D. pluvialis* (89% of visits).

**Table 2:** Consistency and frequency of dominant fly pollinators of the focal annual daisy species.

Color	Plant species	Number of sites	Dominant fly pollinator	Percentage of visits by dominant fly pollinator (mean ± st dev across populations)	Percentage of visits by other pollinator species (range across species)	Percentage of sites with dominant fly pollinator	Percentage of sites with other pollinator species (range across species)
White	*Dimorphotheca pluvialis*	12	*Corsomyza nigripes*	89.0 ± 11.0%	0.1–3.4%	100%	8.3–33.3%
	*Ursinia speciosa*	2	*Corsomyza nigripes*	16.8 ± 6.0%	2.6–36.8%	100%	50%
Orange	*Dimorphotheca sinuata*	37	*Megapalpus capensis*	49.6 ± 32.9%	0.03–7.3%	92%	2.7–40.5%
	*Ursinia cakelifolia*	8	*Megapalpus capensis*	37.3 ± 31.7%	0.2–15.5%	100%	12.5–75%

### To What Extent Do the Distribution Ranges of These Pollinators Overlap?

*Megapalpus capensis* and *C. nigripes* exhibit largely non-overlapping distributions (Figure 2C), and only co-occurred at 7 of 103 survey sites (i.e., 7%). *Megapalpus capensis* was present at low densities at 7 of 15 sites where *C. nigripes* occurred at relatively much higher densities (Figure 2D). Thus the densities of *M. capensis* and *C. nigripes* exhibit opposing geographical patterns across the Namaqualand landscape, with *C. nigripes* occurring at high density in the Sandveld and being absent from the Kamiesberg, while *M. capensis* occurs at highest densities in the Kamiesberg bioregion and is either absent or present at very low densities in the Sandveld (Figures 2C,D).

### Are Flower Color Distributions Associated With Pollinator Availability?

**Figure 3:**
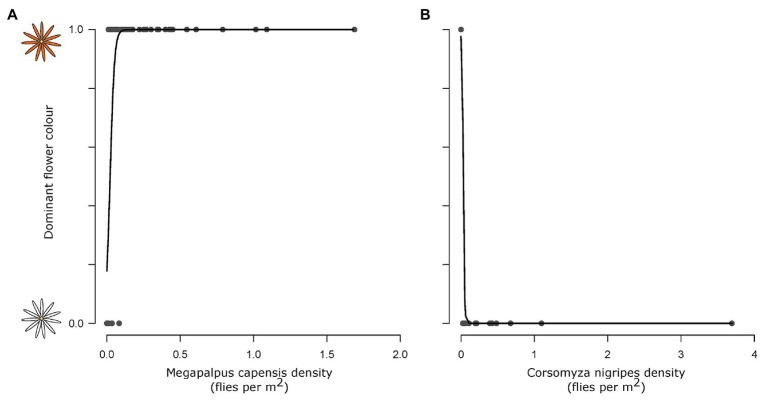
The association between dominant flower color and pollinator species densities. *Megapalpus capensis* **(A)** and *Corsomyza nigripes* **(B)** densities predict flower color of *Dimorphotheca* and *Ursinia* species across 54 sites, where *Megapalpus capensis* is associated with orange flower dominance and *Corsomyza nigripes* is associated with white flower dominance.

The dominant flower color across sites was predicted by fly pollinator densities. High *M. capensis* densities were associated with the presence of orange daisies (*df* = 53, *z* = 2.83, *p* = 0.005, Figure 3), and high *C. nigripes* densities were associated with white daisies (*df* = 53, LR = 41.7, *p* < 0.001).

### Are Flower Colors Detectable and Distinguishable in Fly Vision?

**Figure 4:**
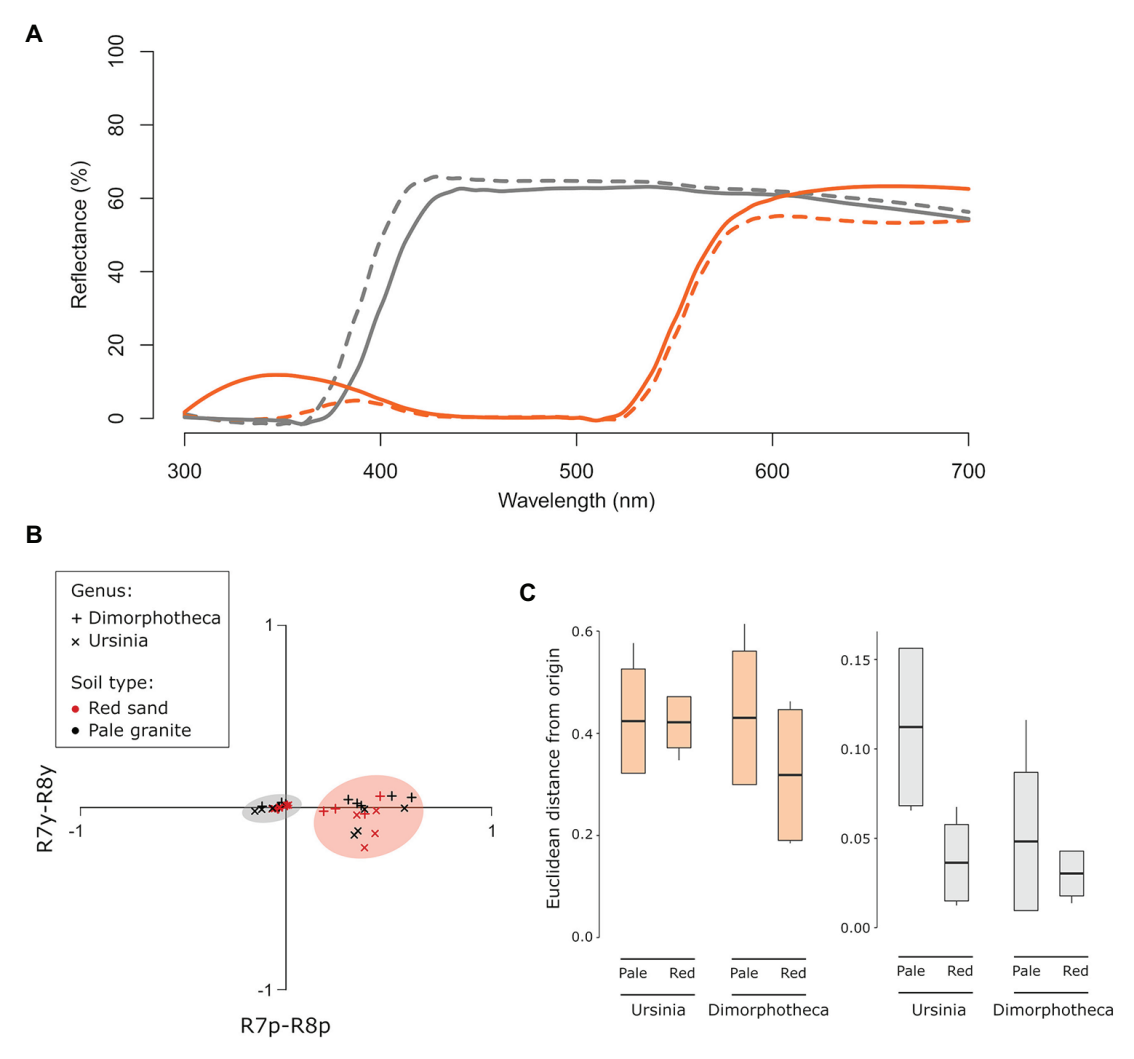
Distinguishability and detectability of daisy flower color in fly vision. Orange flowers are indicated in orange and white flowers in gray throughout. **(A)** Reflectance spectra of ray florets of the four focal daisy species. Dotted lines indicate *Dimorphotheca* species, and solid lines show *Ursinia* species. **(B)** Flower colors modeled in the Troje (1993) fly color space using *Eristalis* receptor sensitivities with adaptation against the alternate soil backgrounds. The orange and gray circles indicate orange and white-flowered species, respectively. **(C)** Difference in detectability, quantified as Euclidean distances from the origin of fly color space, of the orange (*Dimorphotheca sinuata*, *Ursinia cakilefolia*) and white (*Dimorphotheca pluvialis*, *Ursinia speciosa*) species on the two soil backgrounds (pale and red soils). Species did not contrast significantly more strongly against their native soils (pale for orange flowers, red for white flowers) than foreign soils.

Spectra of white-flowered *Dimorphotheca* and *Ursinia* species were very similar, whilst the orange-flowered species did differ in the position and intensity of the secondary short wavelength (UV) peak (Figure 4A). Fly visual modeling suggested that the orange and white flowers can be clearly distinguished from one another, i.e., they fall in separate quadrants of the fly visual model (Figure 4B) and are separated by large color distances (Euclidean distances between centroids of white and orange species: *Dimorphotheca* species on red soil = 0.342, pale soil = 0.471; *Ursinia* species on red soil = 0.442, pale soil = 0.526) that are an order of magnitude larger than the minimum discrimination distances (0.021 in the p−y− quadrant, 0.059 in the p+y− quadrant) suggested by Hannah et al. (2019) for syrphids. In line with reflectance spectra differences, white-flowered species are likely indistinguishable from one another (Euclidean distance = 0.004 on red soil), while the orange-flowered *D. sinuata* and *U. cakilefolia* are more separated in fly color space (Euclidean distance = 0.114 on pale soil, Figure 4B) and tend to occupy different quadrants.

Euclidean distances of orange species from the origin (i.e., background) far exceeded detectability thresholds proposed by Hannah et al. (2019), while those of white species were only marginally greater than the 0.021 unit minimum detectability threshold for syrphids in the green (p−y−) quadrant of fly color space (Figure 4C). This suggests that, on the basis of chromatic information, all species are likely detectable by flies on the soil backgrounds, and that orange species are more detectable against both backgrounds than white species. However, capitula of the same species did not differ significantly in their detectability (i.e., distance from the origin) across the soil backgrounds (*U. speciosa*: *t* = 2.83, *df* = 2.49, *p* = 0.08; *U. cakilefolia*: *t* = 0.06, *df* = 4.36, *p* = 0.96; *D. sinuata*: *t* = 1.30, *df* = 6.62, *p* = 0.24; *D. pluvialis*: *t* = 1.00, *df* = 4.83, *p* = 0.36, Figure 4C), contrary to the expectation that species are more detectable on their native soils.

### Do Pollinators Have Divergent Flower Color Preferences?

**Figure 5:**
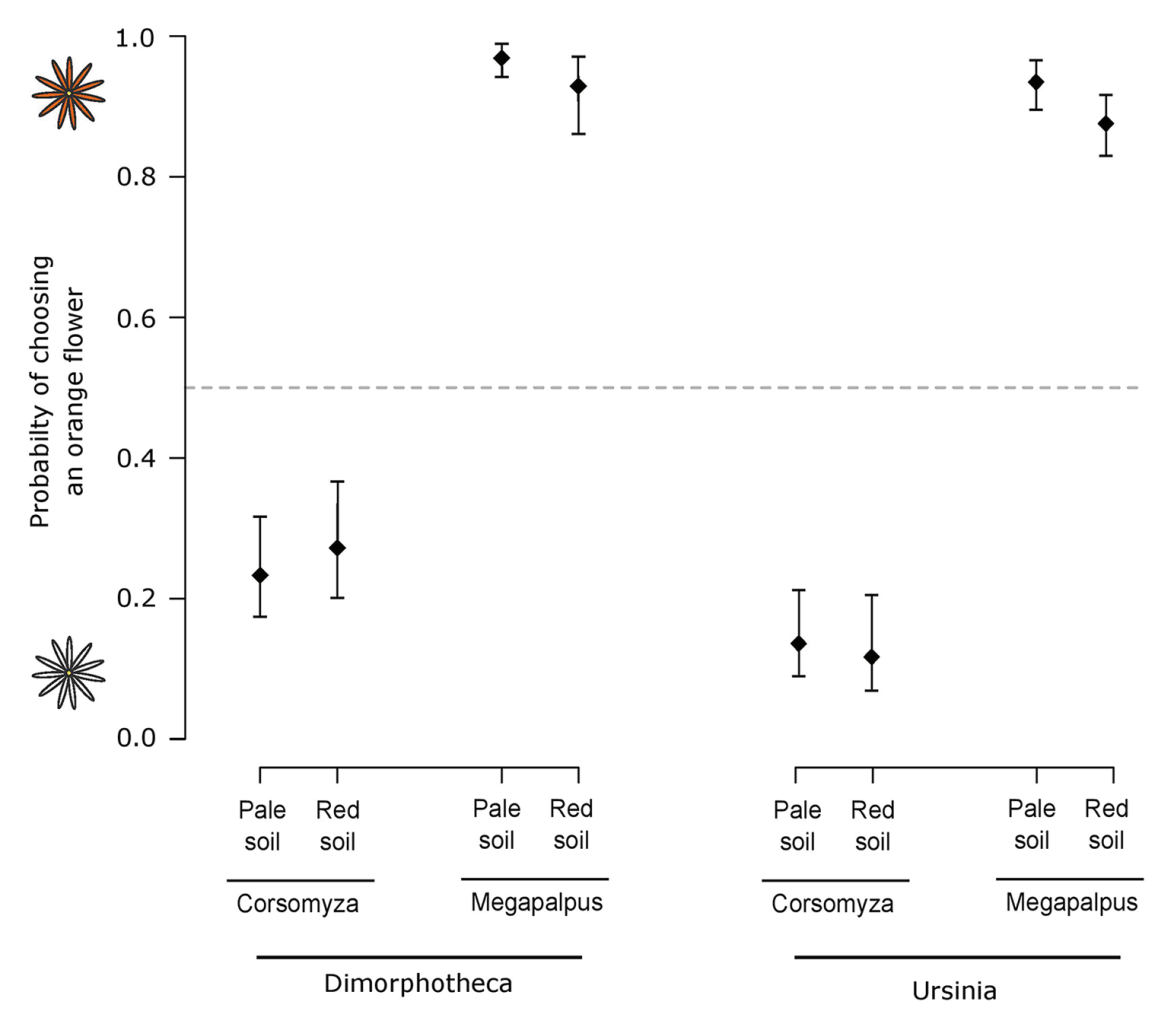
The probability of pollinating flies (*Corsomyza nigripes* and *Megapalpus capensis*) choosing an orange flower over a white flower. Experiments tested fly color preferences using orange-white species pairs of *Dimorphotheca* (orange *Dimorphotheca sinuata* vs. white *Dimorphotheca pluvialis*) and *Ursinia* (orange *Ursinia cakilefolia* vs. white *Ursinia speciosa*) presented on both red and pale soils. *Megapalpus capensis* preferred orange flowers and *Corsomyza nigripes* preferred white flowers of both daisy genera, irrespective of the background-color.

For experiments conducted using *Ursinia*, 47 *M. capensis* individuals made 482 choices and 20 *C. nigripes* individuals made 131 choices. Fly species exhibited different color preferences (Wald = 136.56, *p* < 0.001, Figure 5), with *M. capensis* preferring orange and *C. nigripes* preferring white, and this was not influenced by soil type (Wald = 0.16, *p* = 0.69) or the interaction between insect species and soil type (Wald = 1.10, *p* = 0.30). Similar results were found when doing the experiments using *Dimorphotheca*, with 26 *M. capensis* making 316 choices and 26 *C. nigripes* making 177 choices. Pollinators showed the same divergent color preferences as with *Ursinia* (Wald = 119.99, *p* < 0.001), which were not influenced by soil type (Wald = 0.50, *p* = 0.48) or the interaction between pollinator species and soil type (Wald = 2.91, *p* = 0.09).

**Figure 6:**
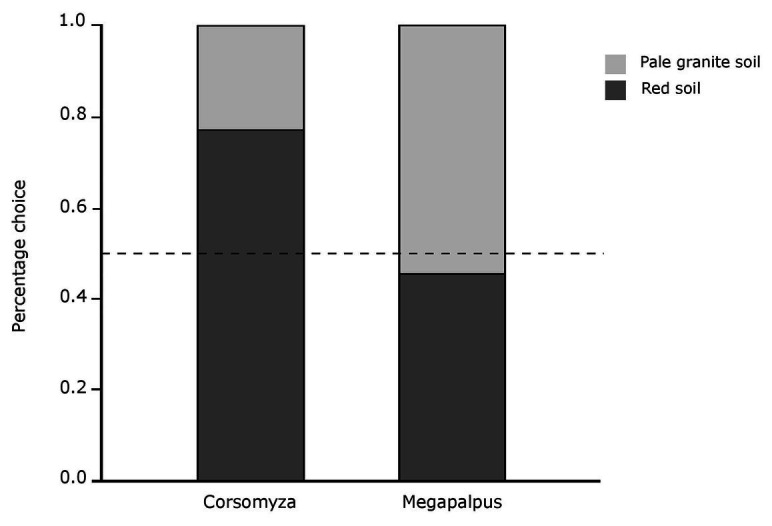
Detectability of flower color on different background soils as quantified by initial choices of fly pollinators for flowers presented on different soil backgrounds. *Corsomyza nigripes* (usually found in white floral communities on red soils) chose white flowers (*Dimorphotheca pluvialis*) on a red soil background significantly more frequently that on pale soil, while *Megapalpus capensis* (usually found in orange floral communities on pale soils) visited orange flowers (*Dimorphotheca sinuata*) on both backgrounds with equal frequency. The dashed line indicates the expected choice ratio if there is no difference in detectability across backgrounds.

*Corsomyza nigripes* flies more frequently chose flowers on red marine-derived soils when allowed to choose between white *D. pluvialis* flowers on the two different soil backgrounds (*χ*^2^ = 9.32, *p* = 0.002, Figure 6). In contrast, *M. capensis* flies showed no preference when allowed to choose between orange *D. sinuata* flowers on different soil types (*χ*^2^ = 0.27, *p* = 0.60, Figure 6).

**Figure 7:**
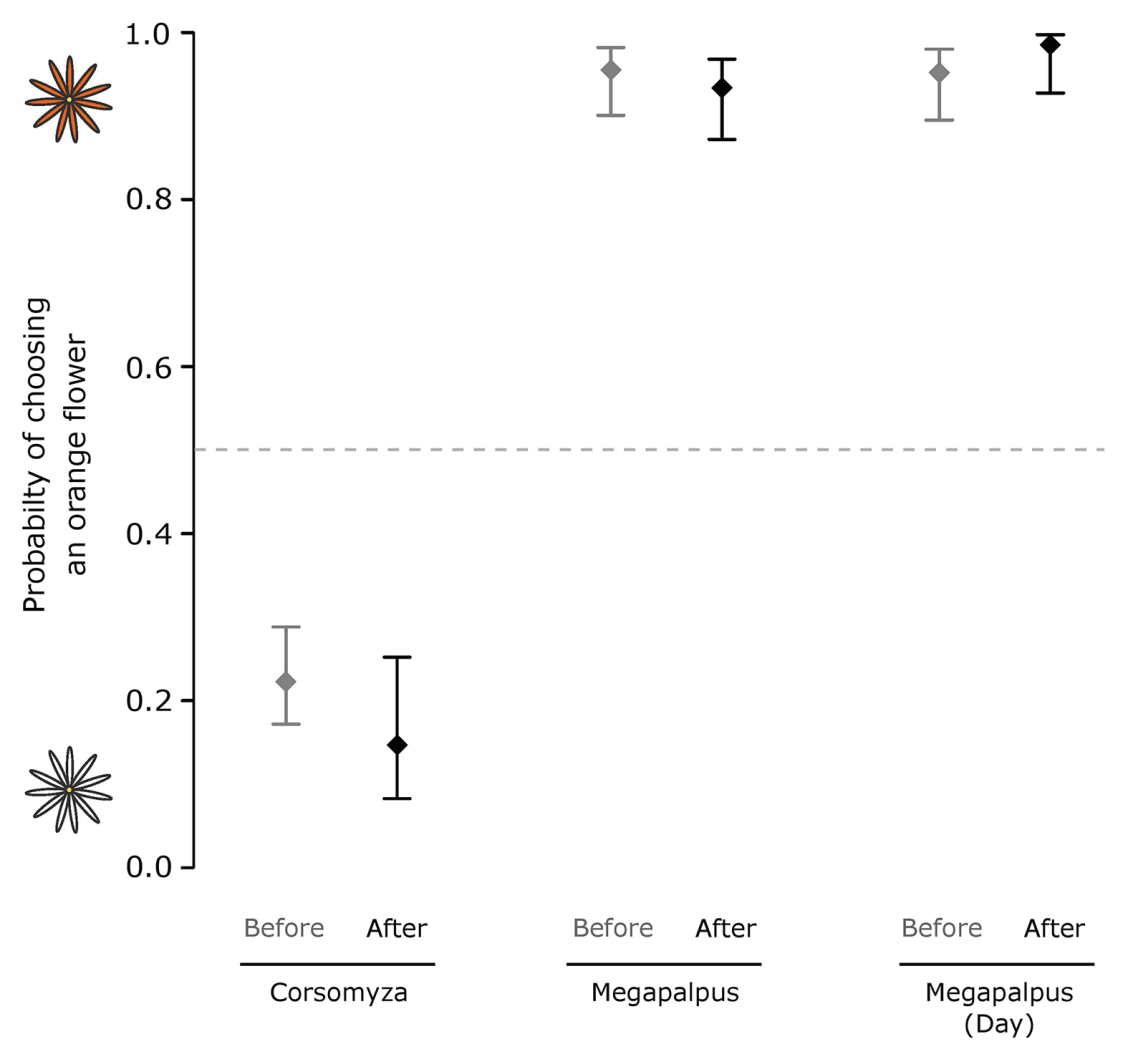
The effect of color conditioning on the flower color choices of two dominant fly pollinator species. Choice experiments were used to characterize flower color choices before and after conditioning. In the first experiment *Corsomyza nigripes* and *Megapalpus capensis* flies were conditioned on flowers of their non-preferred color for 1h, and in a second experiment, *Megapalpus capensis* flies were forced to feed on non-preferred white flowers for a day. Flower color preferences were not altered by conditioning.

Neither pollinator species exhibited altered choices after conditioning on their non-preferred flower color for an hour (*Corsomyza*: Wald = 2.96, *p* = 0.09; *Megapalpus*: Wald = 0.68, *p* = 0.41, Figure 7). *Megapalpus capensis* also did not show a change in preference after feeding on white flowers for a day (Wald = 1.57, *p* = 0.21).

## Discussion

We show that there are steep clines in the spatial turnover of flower color in the annual daisy species that dominate spring flowering displays in Namaqualand, and that change in dominant community flower color is underlain by strong gradients in the density of the dominant fly pollinators in the system. White-flowered species occupy parts of the landscape where *C. nigripes* densities are high and *M. capensis* densities low, while orange flowers dominate when *M. capensis* is present at high densities, but *C. nigripes* is absent. Visual modeling suggested that the orange and white flower colors are distinguishable in fly vision, and flower choice experiments revealed consistently strong and divergent color preferences for orange flowers by *M. capensis* and white flowers by *C. nigripes*. These spontaneous preferences could not be altered by conditioning. While we found some evidence, from choice experiments, that detectability of flowers is influenced by background soil colors, contrast between background and floral color did not alter the direction of strong color preferences. Together these lines of evidence suggest that spatial differentiation of dominant flower colors of annual daisy communities most likely results through selection or filtering imposed by divergent, apparently innate, color preferences across the largely non-overlapping distributions of dominant fly pollinators.

### Color Preferences of Fly Pollinators

Color preferences have been demonstrated for many flies (Lunau, 2014), although seldom in the behavioral context of flower visitation (e.g., Campbell et al., 2010; Lunau et al., 2018; Whitehead et al., 2019). When viewed against the strength of color discrimination and preference quantified from other experimental studies of flies (e.g., Troje, 1993; Campbell et al., 2010; Lunau et al., 2018; Whitehead et al., 2019), the preference of *C. nigripes* for white flowers (80%) and *M. capensis* (91%) for orange flowers (Figure 5) is strong and unambiguous. While we cannot exclude the possibility that choices were influenced by traits other than flower color, the fact that color choices were consistent across two closely related plant species pairs from different genera argues against this possibility. Further, the hues involved are distinguishable under existing models of fly vision (Figure 4), and thus flower color presents a likely cue by which these plant species pairs could be discriminated by fly pollinators in our choice experiments. Given that the Troje (1993) categorical model of fly vision is unlikely to be universally applicable across the diverse visual systems of flies (Lunau, 2014; Hannah et al., 2019; Schnaitmann et al., 2020; van der Kooi et al., 2021), it is perhaps surprising that insights into color discrimination and detectability from our experimental and visual modeling approaches are so well aligned. Whitehead et al. (2019), for example, showed experimentally that *Prosoeca ganglbaueri* (Nemestrinidae) discriminates strongly between colors that are not distinguishable under the categorical fly vision model. Certainly, more research on the visual systems of important pollinating fly lineages, such as the Mariobezziinae, is required to further our understanding of their influence on floral trait evolution.

The strongly divergent color preferences demonstrated here could reflect innate differences in the visual systems of these flies, or alternatively, they could arise because the wild-caught flies used in our experiments were conditioned on the flower colors with which they are associated. As flower-visiting insects, including flies, are frequently able to learn chromatic cues (Dukas, 2008; Lunau et al., 2018), and mate-searching *M. capensis* males quickly learn to avoid female-mimicking floral ornaments (de Jager and Ellis, 2014a), we anticipated that our experimental results might reflect learning. However, we were unable to alter preferences through both short (1h) and long (1day) term conditioning experiments, suggesting that the preferences we observe are spontaneous and not learned. While divergent spontaneous preferences are likely to reflect differences in the innate properties of the visual systems of *C. nigripes* and *M. capensis*, further experiments with naïve individuals, which are currently impossible as the life-histories of these flies are unknown, would be required to confirm this.

The background against which visual floral signals are viewed can influence both their detectability and discrimination (Bukovac et al., 2017). Because the parallel spatial gradients in flower color and pollinators highlighted here correspond to chromatically different soil backgrounds, we explicitly considered this neglected aspect of visual signaling in our experimental and modeling approaches. While visual modeling suggested that apparency of flowers did not differ across soil backgrounds (Figure 4C), choice experiments showed that white *Dimorphotheca* flowers are more detectable to *C. nigripes* on the red sand background, but that detectability of orange flowers did not differ across backgrounds for *M. capensis* (Figure 6). This apparent contradiction between modeling and experimental results may reflect the lack of information about photoreceptor sensitivities of the flies studied here, or general uncertainty in our understanding of fly color vision (van der Kooi et al., 2021). For example, recent work in *Drosophila* demonstrates the importance of inter-ommatidial opponency mechanisms in addition to the intra-ommatidial opponency pathways that underly existing fly color vision models (Heath et al., 2020). Alternatively, flies may use an achromatic processing mechanism (Lunau, 2014), to discriminate bright white flowers against the brighter granite and darker red soil backgrounds. Importantly, despite the influence of background coloration on detectability, strong spontaneous color preferences persist regardless of the background on which flowers were presented. Thus, while background coloration may influence signaling, and should be considered more explicitly in studies of flower color (Bukovac et al., 2017), it does not explain the landscape-scale color patterns described in this manuscript.

### Fly Pollinator Mosaics

Our dataset is novel in that it estimates the “true” underlying pollinator gradient independent of the focal plant species and their phenotypic traits, something which is rarely done (e.g., Phillips et al., 2015). Because Mariobezziinae flies spend the vast majority of their time in flowers (AGE pers. obs.), by surveying all plant species within communities with which they associate (regardless of flower color), our survey approach provides a reliable estimate of fly densities that is independent of the focal plant species. The cause of these pollinator gradients is currently unclear and they could reflect abiotic (e.g., temperature) requirements (Hodkinson, 2005; Inouye et al., 2015) or host requirements of the parasitic larval stages of the bombyliid life-cycle (Yeates and Greathead, 1997). Further work is sorely needed to reveal the drivers of the distributions of these keystone fly pollinators that shape the pollinator climate across the greater cape floristic region.

In many respects, the strong pollinator gradients shown here are unexpected for plants such as daisies with a generalist pollination phenotype (i.e., open access flowers – Ollerton et al., 2007). The gradients that underlie floral differentiation of generalist plants are usually thought to be more nuanced, comprising subtle quantitative differences in the relative availability of different pollinator species (or even functional types) in an assemblage of visitors that remains qualitatively largely unchanged across space (adaptive wandering – Thomson and Wilson, 2008; Gómez et al., 2014). In contrast to this model, the pollinator mosaic that we have quantified here is essentially qualitative, with near-complete turnover in the most abundant daisy visiting fly pollinator species (within the same pollinator functional group) across different habitats in the landscape. This is also true of the other common daisy visiting insect group in Namaqualand, the monkey beetles (Scarabaeidae, Rutelinae, Hoplinii). Monkey beetles also show strong species turnover, and evidence of variable color preferences and flower color associations, across habitat boundaries (Picker and Midgley, 1996; Colville et al., 2002; van Kleunen et al., 2007).

These strong pollinator gradients are also the likely driver of the surprisingly strong ecological specialization (i.e., interactions dominated by few pollinator species) of Namaqualand daisies described here and previously (Ellis and Johnson, 2009; Kemp et al., 2019), despite their generalist phenotypes (i.e., easily accessible pollen and nectar rewards). Because assemblages of potential daisy visitors in communities are dominated by a single abundant, highly-mobile fly species (densities were as high as 1.7 fly individuals m^−2^ for *M. capensis* and 3.7m^−2^ for *C. nigripes*), these species dominate pollinator interactions, resulting in apparent (ecological) specialization of the plants. This effect is not contingent on floral traits that filter less effective pollinators, but results from the strong underlying pollinator density gradients. However, we cannot exclude the possibility that traits, such as pollen/nectar chemistry or disk floret tube lengths, are acting as filters, and thus contributing to pollination specialization, in Namaqualand daisies, something that is in fact suggested by the virtual absence of bees as pollinators (Table 1).

Ecological specialization sets up the potential for strong consistent divergent selection on floral traits, such as color in this case, in these generalist plants across gradients of turnover in dominant pollinators. Thus, these systems are potentially functioning more like the qualitative gradients underlying pollinator-shift models of divergence in phenotypically more specialized plants (Johnson, 2010). Our results suggest that pollinator-shift driven floral differentiation in space is not the sole provenance of pollination specialists, but is also expected in generalists whenever underlying pollinator gradients are strong and spatiotemporally stable enough to create a situation of ecological specialization despite phenotypic generalization.

### Implications for Flower Color Differentiation

While our study convincingly demonstrates that a strong qualitative gradient of spatial turnover in dominant fly species underlies the spatial separation of orange and white-dominated daisy communities because of their divergent color preferences, the mechanistic link between these congruent spatial mosaics remains unclear. On the one hand, pollinator mediated divergent selection may have powered divergence of flower color across the gradient, or geographic variation in color preferences of dominant flies may act as an ecological filter, determining which flower colors are able to establish at different sites. Alternatively, it is also possible that fly color preferences have evolved across gradients of daisy flower color, or that fly communities are ecologically filtered by flower color. While further work is required to resolve this chicken or egg dilemma, some lines of evidence argue against the latter. First, the daisy clades involved are likely more recently evolved than the flies. The genus *Dimorphotheca* is estimated to have arisen 20.12 (8.94–27.88) mya (Barreda et al., 2015), and thus the sister species pair we studied is undoubtedly much younger, as the only taxonomically useful trait separating them is flower color (Norlindh, 1943). In contrast, the split between *Megapalpus* (a monospecific genus) and its sister genus, *Corsomyza*, is substantially older (26 my – de Jager and Ellis, 2017, 38 my – Li et al., 2020). This arguably makes it more likely that flower colors have diverged across a pre-existing geographic mosaic of fly species. Secondly, as *M. capensis* readily fed on white flowers when orange flowers were unavailable in the learning experiments, and is the primary pollinator of a guild of white-flowered pelargoniums in the southern part of its distribution (Struck, 1997), it is unlikely that flower color is limiting the distribution range of *M. capensis*. Regardless, the strong contemporary pollinator mosaic we demonstrate here is likely powering present-day flower color evolution and/or assembly. This is supported by widespread spatial mosaics of intraspecific color variation of annual, fly pollinated daisy species in Namaqualand (e.g., *G. diffusa* – Ellis and Johnson, 2009, *U. calenduliflora* – de Jager and Ellis, 2014b, *D. pinnatum* – Kemp et al., 2019).

Ours is not the first study to highlight the influence of gradients in the relative availability of fly pollinators on the spatial distribution of flower color. It has long been suggested that the dominance of yellow/white flower colors in high alpine communities reflects the relative importance of flies as pollinators because bees are at low density in these communities (Inouye and Pyke, 1988; Pickering and Stock, 2004; Bergamo et al., 2018). Similarly, a recent analysis of flower color on Macquarie Island, where bees are absent and flies dominate pollinator assemblages, showed an unusual prominence of cream-green flowers, that was attributed to filtering of plant colonists by fly pollinators (Shrestha et al., 2016). In both these examples, the assumption is that flies exert significant selection on flower color in generalist plants only when bees (which are assumed to be more effective pollinators because of strong flower constancy – Shrestha et al., 2016) are absent or of reduced importance. Interestingly, this assumption certainly holds in our study system, where bees are virtually absent as pollinators. This does not reflect their absence from the landscape though – bees are diverse and abundant in these Namaqualand communities (Kuhlmann, 2009) – but likely results because the daisies we studied produce very little nectar in comparison to the surrounding plant community, or are actively filtering bees through other means. Intriguingly, because bees have conserved visual systems and exhibit flexible responses to flower color (Chittka et al., 1999; van der Kooi et al., 2021), spatial gradients in the identity of available bee pollinators would not be expected to result in divergent selection on flower color or ecological sorting on the basis of flower color, as is implied by our results. Thus, the striking landscape-level divergence in dominant flower colors that we have investigated here, and which has been demonstrated more widely by Kemp et al. (2019), might well be facilitated by the reliance of these annual daisies on fly pollinators.

## Data Availability Statement

The raw data supporting the conclusions of this article will be made available by the authors, without undue reservation.

## Author Contributions

AE and BA conceptualized the study. AE, JK, and BA collected the data. JK conducted the analyses with input from AE. AE and JK wrote the manuscript with input from BA. All authors contributed to the article and approved the submitted version.

### Conflict of Interest

The authors declare that the research was conducted in the absence of any commercial or financial relationships that could be construed as a potential conflict of interest.
