# Supplementary material for: Geographic Mosaics of Fly Pollinators With Divergent Color Preferences Drive Landscape-Scale Structuring of Flower Color in Daisy Communities
Source: Front Plant Sci. 2021 Feb 1;12:617761. doi: 10.3389/fpls.2021.617761 (PMC7882612; doi:10.3389/fpls.2021.617761)
Supplement: Supplementary file 1 [file Data_Sheet_1.pdf]

Supplementary file: Ellis, Anderson, Kemp.

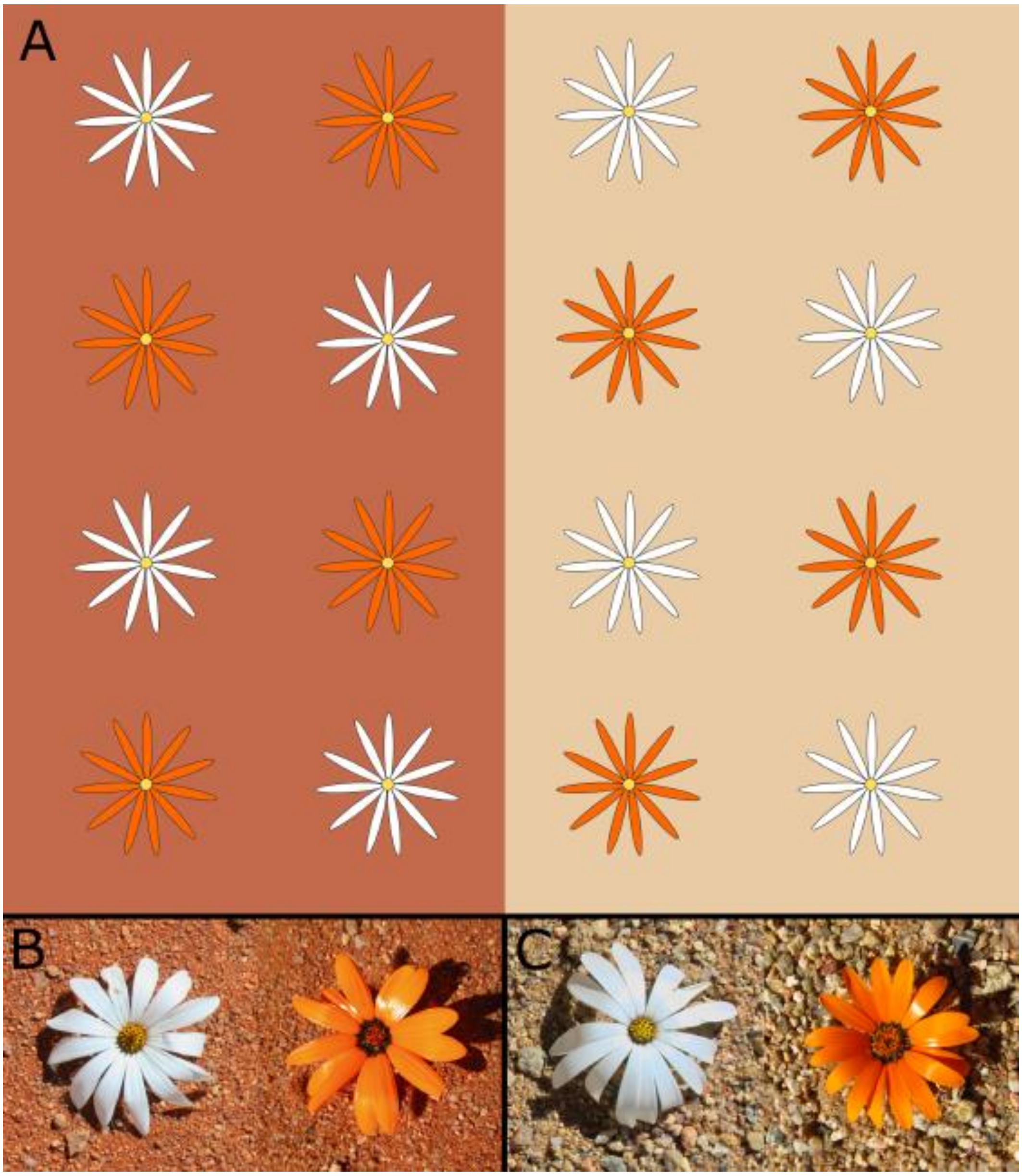

Figure S1. The experimental setup for flower colour choice experiments. (A) Eight white and eight orange inflorescences were presented on two soil types (red marine-derived soils – B; and pale granite gneiss-derived soils – C). Bee fly individuals were offered choices of orange-white species pairs of *Dimorphotheca* and *Ursinia* separately.
